# An Improved Imaging Algorithm for High-Resolution Spotlight SAR with Continuous PRI Variation Based on Modified Sinc Interpolation

**DOI:** 10.3390/s19020389

**Published:** 2019-01-18

**Authors:** Shiyang Chen, Lijia Huang, Xiaolan Qiu, Mingyang Shang, Bing Han

**Affiliations:** 1 Institute of Electronics, Chinese Academy of Sciences, Beijing 100190, China; chenshiyang16@mails.ucas.ac.cn (S.C.); xlqiu@mail.ie.ac.cn (X.Q.); shangmingyang16@mails.ucas.edu.cn (M.S.); han_bing@mail.ie.ac.cn (B.H.); 2 Key Laboratory of Technology in Geo-Spatial Information Processing and Application System, Institute of Electronics, Chinese Academy of Sciences, Beijing 100190, China; 3 School of Electronic, Electrical and Communication Engineering, University of Chinese Academy of Sciences, Beijing 100049, China

**Keywords:** modified sinc interpolation, spotlight SAR, PRI variation, high-resolution

## Abstract

This paper focuses on an improved imaging algorithm for spotlight synthetic aperture radar (SAR) with continuous Pulse Repetition Interval (PRI) variation in extremely high-resolution. Conventional SAR systems are limited in that a wide swath cannot be achieved with a high azimuth resolution in the meantime. This limitation can be overcome by Pulse Repetition Frequency (PRF) variation in a SAR system. However, there are problems such as the ambiguities of point targets or extended targets caused by nonuniform sampling. A reconstructive method, Nonuniform Discrete Fourier Transform (NUDFT) has been presented in the current literature, but it is rather computationally expensive. In this paper, a modified sinc interpolation based on NUDFT is proposed, which is used to reconstruct the uniformly sampled echo in time domain. Since the interpolation kernel length is relatively short, it is more computationally efficient. Then, the two-step processing approach combined with the modified sinc interpolation is further presented, which has much better accuracy than that combined with the conventional sinc interpolation. Both the simulated data and the extracted GF-3 data experiment demonstrate the validity and accuracy of the proposed approach.

## 1. Introduction

Synthetic aperture radar (SAR) is a well-proven remote sensing technique for high-resolution imaging of the Earth’s surface, while it is independent of weather and illumination conditions. Swath width and resolution are two important performance specifications in the SAR system. However, the requirements of wide-swath imaging and high azimuth resolution cannot be satisfied in the meantime in the SAR system design. To control range ambiguities, the PRI must be larger than the time that it takes to collect returns from the entire illuminated swath which result in a lower bound of pulse repetition frequency (PRF). On the other hand, to avoid significant azimuth ambiguity levels, a large PRI or equivalently a low PRF implies the adoption of a small Doppler bandwidth and limits the achievable azimuth resolution. This has encouraged lots of research to focus on resolving the limitation of wide swath and high azimuth resolution [1,2,3,4]. In the conventional SAR systems with a fixed PRF, stationary blind ranges occur when the echo arriving time coincides with the pulse transmitting time, hence blocking the continuous reception of pulses. In spotlight mode, the transmit interference aggravates as the range migrates in longer synthetic aperture time, and thus there could be no acceptable PRF for a given swath width, especially for high squint angle and high look angle. An alternative approach to settle the problems mentioned above (fixed blind ranges and the contradiction of resolution and swath) is the periodic linear variation of the pulse repetition interval (PRI). As the PRI varies, the blind ranges change along with the azimuth time, and the sequence of the received signal of every target can be mostly acquired. This PRI variation technique can achieve continuously ultrawide swath imaging without azimuth resolution loss in company with the digital beam-forming (DBF) technique [5,6,7].

The design of a PRI sequence with slow and fast change is analyzed in Ref. [8]. The principle of slow changing sequences of PRIs is to minimize the PRI span such that the blind ranges are almost uniformly spread over the slant ranges of the illuminated area. As for fast PRI variation, the principle to design sequences of PRIs is to avoid missing two consecutive samples in the raw azimuth signal for all slant ranges of interest. In order to eliminate the effects caused by nonuniform sampling in azimuth direction, like spectrum distortion and false targets, many reconstruction methods have been proposed, such as back projection (BP) algorithm [6,7], multichannel reconstruction, linear interpolation, best linear unbiased (BLU) interpolation [8], etc. Although the BP algorithm is available [9,10], it suffers from significant computational complexity due to the point-by-point correlation in the time domain. Besides, as the received azimuth signal is not strictly bandlimited, a reconstruction error will be present when the multichannel reconstruction is employed [11,12], as the signal components outside the aforementioned frequency band fold back to the main part of the spectrum and disturb the reconstruction of the signal itself. The BLU interpolation requires a large oversampling factor and many computational resources. In this paper, a modified sinc interpolation based on Nonuniform Discrete Fourier Transform (NUDFT) [13] is proposed. It can be directly combined with the traditional two-step processing approach to imaging the SAR echo in spotlight mode with high-resolution and PRI variation. Simulation results demonstrate that the modified sinc interpolation has almost the same performance as that of NUDFT, meanwhile, it reduces the computational cost efficiently.

This paper is organized as follows: In Section 2, the design of the variant PRI sequence with slow change and fast change are discussed. The parameters of them are set and then the blind ranges distributions are analyzed. In Section 3, the modified sinc interpolation is deduced out based on NUDFT, and then the two-step processing algorithm combined with the modified sinc interpolation is given for the variant PRI case. In Section 4, the performances are assessed both by the simulated data of the point target and by the extracted GF-3 data. In Section 5, the conclusion of the whole paper is given.

## 2. PRI Variation

### 2.1. Fixed PRI

The Conventional SAR systems usually employ a fixed PRI, or equivalently a fixed PRF. The value of PRF is restricted by many factors of the conventional SAR system. The azimuth ambiguity-to-signal-ratio (AASR) and range-ambiguity-to-signal-ratio (RASR) are highly related to the PRF value. A low value of PRF increases the AASR due to the azimuth spectrum aliasing effect. On the other hand, a high PRF value will reduce the interpulse period and result in overlap between the received pulses, thus leading to an increase of RASR. Furthermore, the PRF selection is constrained for a conventional SAR system because there is a single antenna for both transmitting and receiving processes. Also, the PRF is selected to ensure that the nadir return from succeeding pulses is excluded from the echo recording window, because the nadir return leads to possible saturation effects in imaging processing.

The PRF restriction of transmit interference can be defined as follows:

(1) $$2R_{\max}/c-1/prf+\tau_{g}<i/prf<2R_{\min}/c-\left(T_{r}+\tau_{g}\right)$$

where $R_{\min}$ and $R_{\max}$ are the minimum and maximum slant range of the interested area, c represents light speed, prf is the fixed PRF, $T_{r}$ is the transmitting pulse duration, and $\tau_{g}$ is time of the receiver protect window extension, and i=0,1,2,… denotes the number of non-negative integer data sampling points.

The range ambiguity restriction on PRF can be written as follows:

(2) $$\left(2R_{\max}/c\right)-2T_{r}-\left(1/prf\right)<\left(2h/c\right)+\left(j/prf\right)<\left(2R_{\min}/c\right)$$

where h is the sensor altitude above the surface nadir point and j=0,1,2,… means non-negative integer.

The span of acceptable PRF values is, therefore, established by the aforementioned analysis. In general, with the off-nadir angle increasing, the available PRF span is reduced and the ambiguity-to-signal ratio is lowered. In the spotlight SAR, as the resolution and the off-nadir angle increase, the synthetic aperture time enlarges as well as the range migration amount. Therefore, the imaging-swath is further reduced, and there could be no acceptable PRF in a certain condition. 

**Figure 1:**
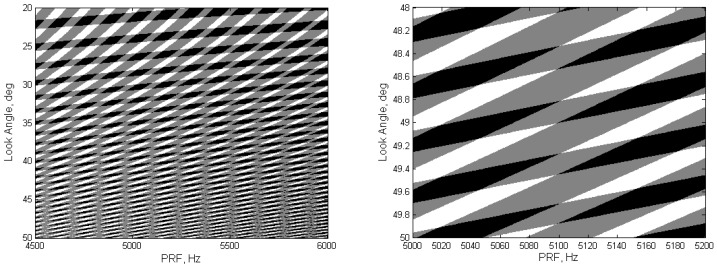
Illustration of PRF excluded zones as a result of transmit and nadir interference. (**a**) PRF: 4500 Hz–6000 Hz, look angle: 20°–50°; (**b**) PRF: 5000 Hz–5200 Hz, look angle: 48°–50°.

As an example, for spaceborne SAR with spotlight mode, the sensor altitude is 1100 km, and the requirement of resolution and swath is 0.1 and 8 km × 8 km respectively. The excluded zones of PRF choice defined by Equations (1)–(2) are shown in Figure 1a, where the PRF span is 4500 Hz to 6000 Hz and the look angle ranges from 20° to 50°. By increasing the look angle to around 49°, there is no acceptable PRF inside the range from 5000 Hz to 5200 Hz, as shown in Figure 1b to get the imaging-swath wider than 6 km. So, it is difficult for the conventional SAR with fixed PRF to meet both the high-resolution and the wide-swath requirements at the same time.

### 2.2. Periodically Varied PRI

Through the periodical PRF variation, the blind ranges are no longer fixed but uniformly distributed in the whole imaging swath. Therefore, the transmit and nadir interference are no longer constraints in the SAR system. Then, it is possible to achieve a high-resolution and wide swath by employing the PRF variation technique. Two types of PRI sequence can be chosen according to system demands, and the approach of establishing parameters for PRI sequences of slow change and fast change will be discussed in the following.

#### 2.2.1. Slow Linear Variation 

The criterion of slow linear variation of the PRI sequence design is to minimize the PRI span to reduce the effect on range and azimuth ambiguities. To make the blind ranges distribute uniformly in the interested illuminated area, the acceptable PRF span between $PRF_{\min}$ and $PRF_{\max}$ is constrained by the following equation:

(3) $$PRF_{\max}-PRF_{\min}\geq c/2R_{\max}$$

where $R_{\max}$ should be given initially according to the system demand of the imaging swath; $PRF_{\min}$ needs to satisfy the Nyquist sampling theory of Doppler bandwidth; then $PRF_{\max}$ can be calculated through Equation (3). Finally, the sequence length N and the difference of consecutive PRIs must be chosen such that the sum of N PRIs (the period of one sequence) is approximately one-fifth of the synthetic aperture time at the near range.

#### 2.2.2. Fast Linear Variation

The design of slow linear variation of PRI would result in consecutive pulses losing. The principle to design fast change sequences of PRIs is to avoid missing two consecutive samples in the raw azimuth signal for all slant ranges of interest. According to principle [11], the difference of two consecutive pulses should satisfy the constraint:

(4) $$\Delta \geq \Delta_{\min}=\frac{2T_{r}}{k^{*}}$$

where $k^{*}$ means that the $k^{*}$th pulse is transmitting while the first pulse is received from the slant range $R_{\min}$, Δ is the difference between two consecutive PRIs and $\Delta_{\min}$ is the minimum of the difference. Furthermore, $k^{*}$ is the key factor to avoid consecutive pulses losing [8], and it is defined as:

(5) $$k^{*}=\left\lfloor \frac{\frac{2R_{\min}}{c}+PRI_{0}-2T_{r}}{PRI_{0}-T_{r}}\right\rfloor$$

Finally, the sequence length N can be obtained through the following Equation [8]:

(6) $$N\geq N_{\min}=\left\lfloor \frac{\left(PRI_{0}+\frac{\Delta }{2}\right)-\sqrt{{\left(PRI_{0}+\frac{\Delta }{2}\right)}^{2}-2\Delta \left(\frac{2R_{\max}}{c_{0}}+T_{r}-\Delta +\left(PRI_{0}+\frac{\Delta }{2}\right)k^{*}-\frac{\Delta }{2}k^{*2}\right)}}{\Delta }\right\rfloor$$

where the ⌊⋅⌋ means the function to get the largest integer not greater than the precisely calculated value. Once $PRI_{\min}$ is set according to the Nyquist sampling theory of Doppler bandwidth, the parameters (including $k^{*}$, Δ, and N) of fast linear PRI variation are determined by Equations (4)–(6).

#### 2.2.3. SAR Parameters of Two Types of PRI Variations 

**Figure 2:**
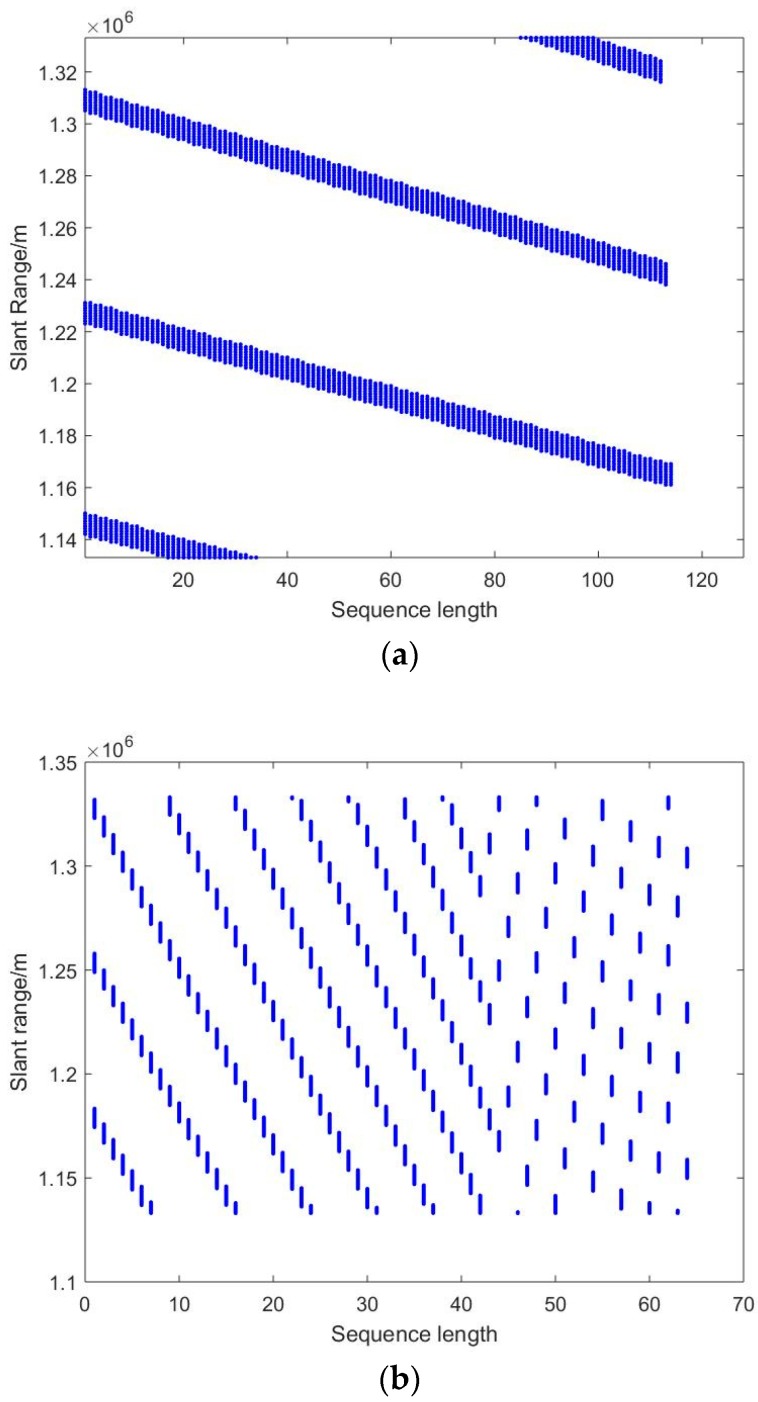
Blind ranges location for a given parameter of different PRI variations: (**a**) Slow linear PRI variation; (**b**) fast linear PRI variation.

The SAR parameters for both the fast variation and slow variation of PRI calculated by the Equations mentioned above are listed in Table 1. According to Table 1, the blind ranges locations are illustrated in Figure 2.

In Figure 2a, the design of slow linear variation of PRI causes the blind ranges to be located uniformly in the imaging area, where the available width is much wider than 8 km. However, there is the problem of a dozen losing pulses for certain slant range. In Figure 2b, the design of fast linear variation of PRI makes the blind ranges distribute more desperately because the span of PRF variation is much larger than that of slow variation. The problem of losing consecutive pulses is settled in the meantime. For both two PRI variation types, the available width is much wider than 8 km if the DBF technique is employed. The type of PRI variation can be chosen according to system demand. 

## 3. Imaging Algorithm for Spotlight SAR with PRI Variation

### 3.1. Modified Sinc Interpolation

A bandlimited continuous signal can be reconstructed by employing a sinc interpolation [14,15,16]. Provided that the Nyquist sampling rate is satisfied, the sampling theorem states that the bandlimited signal g(t) can be reconstructed from uniform samples without error through sinc interpolation as follows:

(7) $$\begin{array}{r} g(t)=\sum_{i}g(\frac{i}{prf})\sin c\left[prf(t-\frac{i}{prf})\right]\\ \approx \sum_{i=0}^{N-1}g(\frac{i}{prf})\sin c\left[prf(t-\frac{i}{prf})\right] \end{array}$$

where prf is the uniform sampling rate and i represents sampling instants where i=0,…,N−1.

However, the nonuniform sampling signal or the non-baseband signal, which is the azimuth signal of SAR with PRI variation, cannot be reconstructed by the traditional sinc interpolation mentioned above. Moreover, the Doppler spectrum of the nonuniform sampling azimuth signal cannot be calculated by Fast Fourier Transform (FFT). A generalization of the Discrete Fourier Transform (DFT), i.e., NUDFT, can be employed to obtain the Doppler spectrum, which is:

(8) $$S_{a}\left(f\right)=\int_{t}s_{a}\left(t\right)e^{-j2\pi ft}dt\approx \sum_{i}s_{a}\left(t_{i}\right)e^{-j2\pi ft_{i}}\Delta t_{i}$$

where $s_{a}\left(t\right)$ and $S_{a}\left(f\right)$ represent the azimuth signal forms in the time and frequency domain respectively, f is the variable of the frequency domain, $t_{i}$ is the nonuniform sampling instants of the *i*th samples, and $\Delta t_{i}=t_{i+1}-t_{i}$.

In terms of the azimuth pattern weighting, the main energy of $S_{a}\left(f\right)$ is confined in the band which is centered at the Doppler centroid frequency (denoted by $f_{dc}$) and bounded by prf. Accordingly, Equation (8) can be rewritten as:

(9) $$S_{a}\left(f\right)\approx \sum_{i}s_{a}\left(t_{i}\right)e^{-j2\pi ft_{i}}\Delta t_{i}\cdot \mathrm{rect}\left(\frac{f-f_{dc}}{prf}\right)$$

Derived from Equation (9), we can obtain that:

(10) $$s_{a}\left(t\right)=prf\sum_{i=0}^{N-1}\left[s_{a}\left(t_{i}\right)\Delta t_{i}\sin c\left[prf\left(t-t_{i}\right)\right]e^{j2\pi f_{dc}\left(t-t_{i}\right)}\right]$$

Comparing Equation (7) with Equation (10), the reconstructed expression of the nonuniformly sampled signal can be obtained as follows:

(11) $$\begin{array}{r} s_{a}\left(t\right)=prf\sum_{i}\left\{s_{a}\left(t_{i}\right)\Delta t_{i}\sin c\left[prf\left(t-t_{i}\right)\right]e^{j2\pi f_{dc}\left(t-t_{i}\right)}\right\}\\ \approx prf\sum_{i=0}^{N-1}\left\{s_{a}\left(t_{i}\right)\Delta t_{i}\sin c\left[prf\left(t-t_{i}\right)\right]e^{j2\pi f_{dc}\left(t-t_{i}\right)}\right\} \end{array}$$

The interpolation kernel is a sinc-like function $\sin c\left[prf\left(t\right)\right]e^{j2\pi f_{dc}t}$ adjusted by the coefficient. For the uniformly sampled signal and baseband signal, we have $\Delta t_{i}=1/prf$ and $f_{dc}=0$; then Equation (11) goes back to Equation (7). Compared with the conventional sinc interpolation, the modified sinc interpolation can be employed to the reconstruction of the bandlimited signal with nonuniform samples.

The tradeoff between accuracy and computational cost must be taken into consideration when selecting the length of the modified sinc interpolation kernel (denoted by *L*). The longer the length *L* is, the better the modified sinc interpolation performs in reconstruction in theory. In practice, an interpolation kernel that uses a large *L* would be very costly. Fortunately, the kernel weight decreases rapidly with the distance away from the interpolation point. Thus, length *L* can be shortened without much loss of accuracy. In the experiments of this paper, the desired results can be obtained while L=64.

The calculating complexity of NUDFT and modified sinc interpolation methods are shown in Table 2, where *Na* is the total of sampling points in azimuth direction. The ratio of calculating the complexity of NUDFT to that of the modified sinc interpolation is (2Na−1)/(2L−1). Since L≪Na, it is obvious that the computational cost of the modified sinc interpolation is far less than that of NUDFT.

### 3.2. Modified Two-Step Processing Approach Based on Modified Sinc Interpolation

By beam steering in the azimuth direction, the spotlight SAR effectively achieves high-resolution images of the illuminated area [17,18,19,20]. The two-step processing approach is widely used in spotlight SAR data focusing. For the spotlight SAR, the azimuth total bandwidth consists of instantaneous Doppler bandwidth and target Doppler bandwidth. In actual SAR systems, PRF is larger than the instantaneous Doppler bandwidth but much less than the azimuth total bandwidth. As a consequence, the data processing procedure in the frequency domain, as that carried out for efficient strip-mode focusing, cannot be directly implemented on the received echo because of the azimuth spectrum folding effect. The key point of the two-step processing approach is the deramping procedure (multiplying the azimuth signal by a properly chosen chirp signal). This procedure is attractive because it has computational efficiency to overcome the azimuth spectrum folding effect. After deramping, the echo turns to be bandlimited which makes the modified sinc interpolation applicable. As mentioned above, the spotlight SAR with PRI variation has advantages in swath width and resolution. To deal with the problems caused by nonuniform sampling, the modified sinc interpolation is employed as a preprocessing procedure, so that the two-step processing approach can still be used. 

The SAR echo backscattered from a point target can be expressed as follows:

(12) $$s(\tau ,\eta )=rect\left(\frac{\tau -2R(\eta )/c}{T_{r}}\right)\exp\left(j\pi k_{r}{\left(\tau -\frac{2R(\eta )}{c}\right)}^{2}\right)\times w_{a}\left(\eta \right)\exp\left(-j\frac{4\pi f_{c}R(\eta )}{c}\right)$$

where τ and η are range and azimuth time, $f_{c}$ is the carrier frequency of the radar, R(η) is the range history from the point target to the radar antenna phase center, rect(⋅) is the rectangular window bounded to the range signal in time domain, and $w_{a}\left(\cdot \right)$ is the window bounded to the azimuth signal in the time domain. The window $w_{a}\left(\cdot \right)$ is affected by azimuth pattern, azimuth beam rotation velocity and synthetic aperture time.

To simplify the discussion, the signal can be transformed into range frequency and azimuth time domain, and then can be defined as follows:

(13) $$S(f_{\tau },\eta )=rect\left(\frac{f_{\tau }}{B_{r}}\right)\exp\left(-j\pi \frac{{f_{\tau }}^{2}}{k_{r}}\right)\times w_{a}\left(\eta \right)\exp\left(-j\frac{4\pi (f_{c}+f_{\tau })R(\eta )}{c}\right)$$

where $f_{\tau }$ is the range frequency.

In the range frequency and azimuth time domain, the signal in each range gate has the form of a chirp-like signal, which is given by:

(14) $$s_{a}(\eta )=w_{a}\left(\eta \right)\exp\left(-j\frac{4\pi (f_{c}+f_{\tau })R(\eta )}{c}\right)$$

To solve the spectrum folding problem, the first step of the procedure involves the azimuth convolution between the echo and the deramping chirp signal. The convolution can be asymptotically evaluated by applying the stationary-phase method, wherein the resulting signal depends on the product as follows:

(15) $$s_{a}(\eta^{\prime })=h(\eta^{\prime })\times FFT(s_{a}(\eta )\times h(\eta ))$$

where h(η) is the chirp signal,$\eta^{\prime }$ is a new azimuth time with upsampling interval.

**Figure 3:**
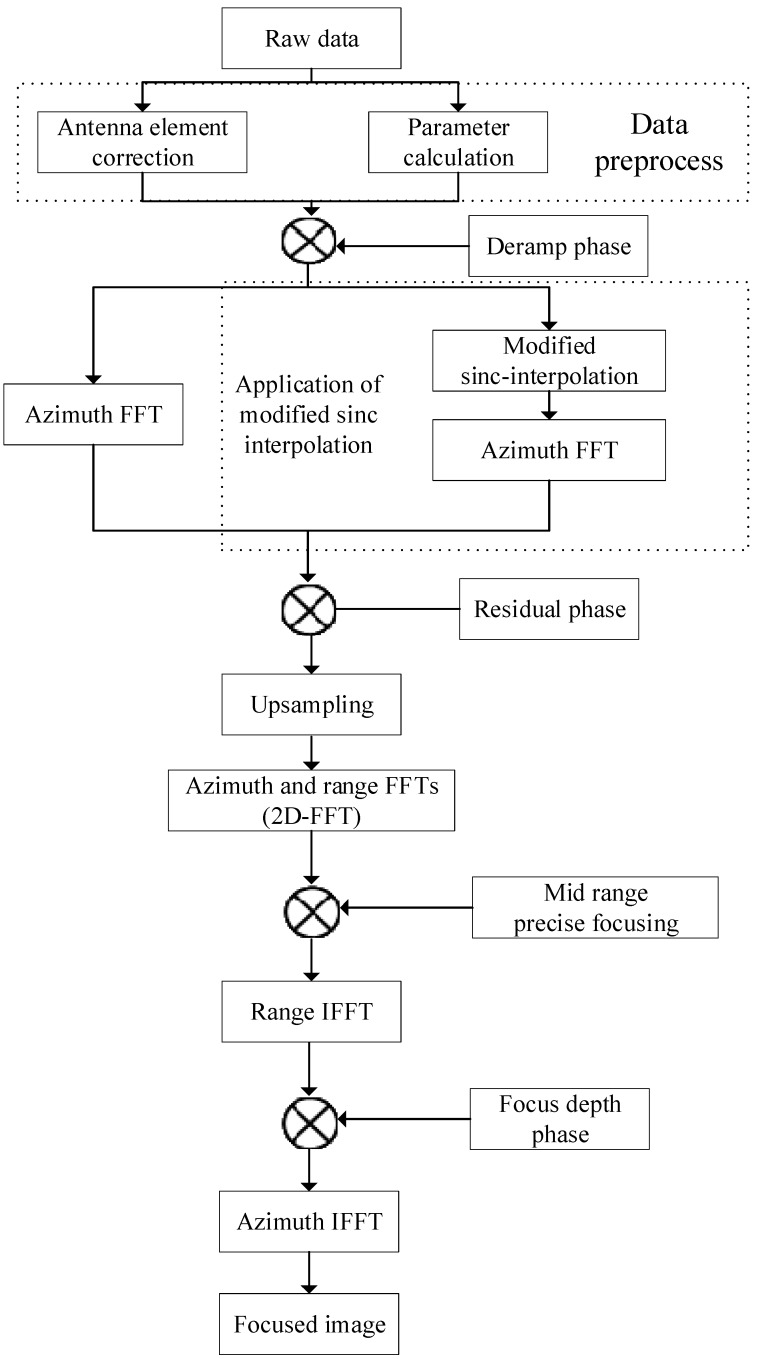
Procedure of the two-step processing approach with the modified sinc interpolation.

Then, the whole imaging processing block diagram is shown in Figure 3.

For the nonuniformly sampled signal, the modified sinc interpolation is needed. After multiplying $s_{a}(\eta )$ with h(η), the azimuth signal turns to be bandlimited, and the modified sinc interpolation can be implemented to reconstruct the uniformly sampled signal after this step. It should be noticed that, the chirp signal is generated with respect to the variant PRI samples.

For spotlight SAR with continuous PRI variance, the deramp procedure of the two-step imaging algorithm is modified as follows:

(16) $$s_{a}(\eta^{\prime })=h(\eta^{\prime })\times FFT(MSINC(s_{a}(\eta )\times h(\eta )))$$

where MSINC(⋅) means the modified sinc interpolation.

After that, the stripmap processing procedure is applied like the conventional two-step processing approach. The whole processing procedure is present in Figure 3. 

## 4. Simulation Results

In order to validate the proposed method, a number of experiments have been carried out using simulated data and GF-3 data. The simulation results of 1 D azimuth signal are shown in this section to compare the accuracy and the efficiency between NUDFT, the conventional sinc interpolation and the modified sinc interpolation method. Furthermore, both of the two different linear sampling methods of fast and slow PRI variation given in Table 1 are analyzed. Three targets are uniformly distributed along the azimuth illuminated scene width (8 km), where the interval is 4 km.

### 4.1. Results of Slow PRI Variation

**Figure 4:**
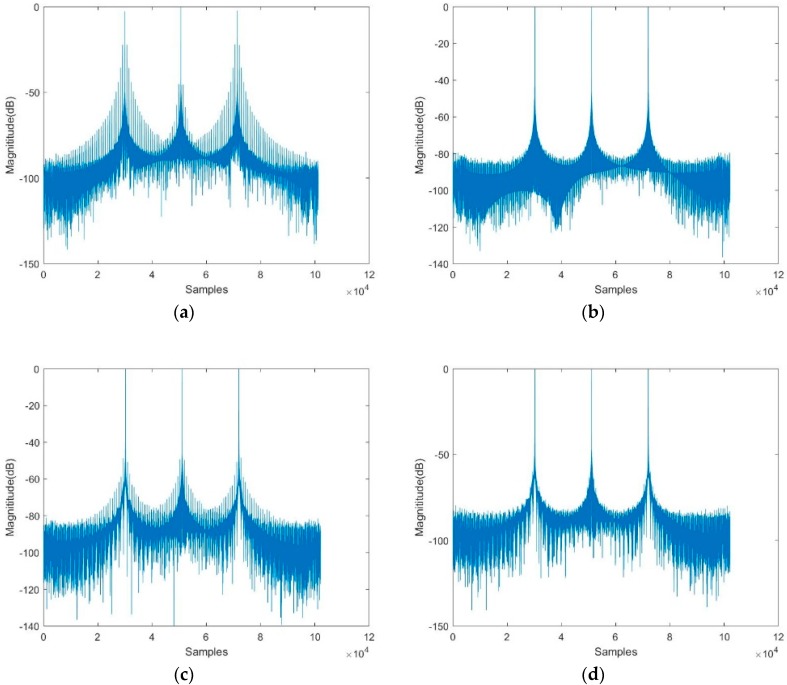
Nonuniform sampled signal with slow PRI change after compression. (**a**) Compression result with FFT. (**b**) Compression result with NUDFT. (**c**) Compression result with sinc interpolation. (**d**) Compression result with modified sinc interpolation.

The simulation results given in Figure 4 are based on the spotlight spaceborne SAR parameters of slow PRI change sequence.

In Figure 4a, the level of the ambiguous targets reaches near −20 dB by the conventional two-step imaging algorithm involving FFT. Besides, as shown in Figure 4c, the two-step imaging algorithm by using the sinc interpolation performs a little better but the level of ambiguous targets is still apparent near −50 dB. In Comparison, Figure 4b shows the compression results from the two-step algorithm in which the azimuth FFT operation is substituted by NUDFT, and Figure 4d shows the results from the modified sinc interpolation combined with the two-step processing algorithm given in this paper. The level of ambiguous targets is efficiently reduced to about −70 dB. Thus, the modified two-step algorithm combined with the modified sinc interpolation is proved to be sufficient for spotlight SAR with slow PRI variation.

### 4.2. Results of Fast PRI Variation 

**Figure 5:**
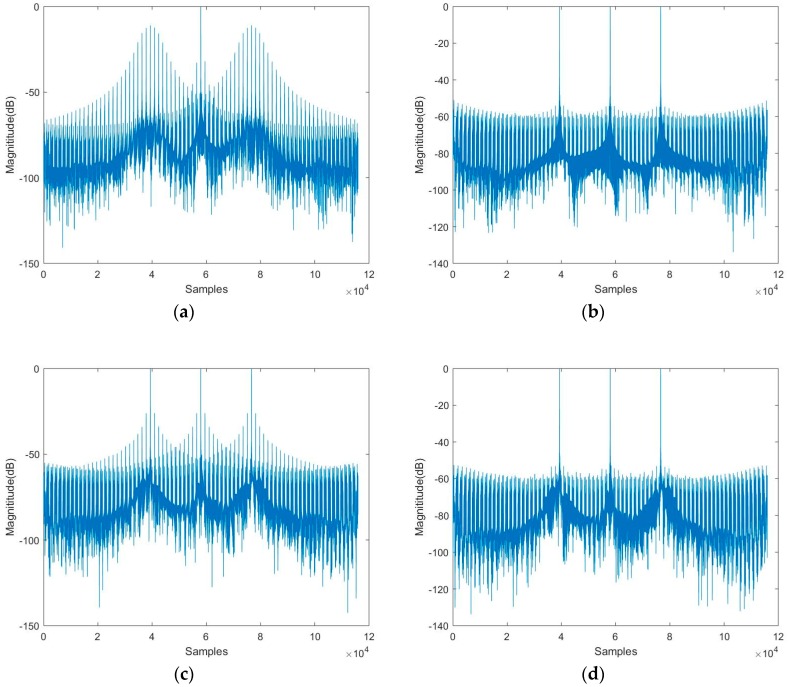
Nonuniform sampled signal with fast PRI change after compression. (**a**) Compression result with FFT. (**b**) Compression result with NUDFT. (**c**) Compression result with sinc interpolation. (**d**) Compression result with modified sinc interpolation.

The simulation results given in Figure 5 are based on the spotlight spaceborne SAR parameters of fast PRI change sequence. 

From Figure 5a, the azimuth echo cannot be properly compressed by the conventional two-step processing approach algorithm involving FFT, the near range target and the far range target almost have sidelobes of the same amplitude as the main lobe. It is also apparent in Figure 5c that high sidelobes are presented near the main lobes in three targets through the traditional sinc interpolation, which is about −26 dB. As far as the modified sinc interpolation or NUDFT is employed, the level of sidelobes is reduced to near −54 dB in Figure 5b,d.

The level of the false targets of the simulation results is summarized in Table 3 for the impulse responses of Figure 4 and Figure 5. As shown in Table 3, the modified sinc interpolation combined with the two-step processing approach effectively reduces the level of false targets with either the fast change PRI variation sequence or slow change PRI variation sequence, compared to the traditional sinc interpolation or FFT involved in the conventional two-step processing approach. 

### 4.3. Experiments on GF-3 Data

To give an idea of the image quality improvement of actual SAR image, a GF-3 data, acquired over the city of Nanjing, China, has been used. As the raw data of GF-3 is uniformly sampled, data-preprocessing is employed to create nonuniformly sampled data [8]. The data-preprocessing is used to lose pulses periodically, which we bend one pulse per five pulses to build the periodical nonuniformly sampled sequences. After data-preprocessing, the equivalent PRF is about 3000 Hz which still satisfies the Nyquist sampling theory of Doppler bandwidth.

**Figure 6:**
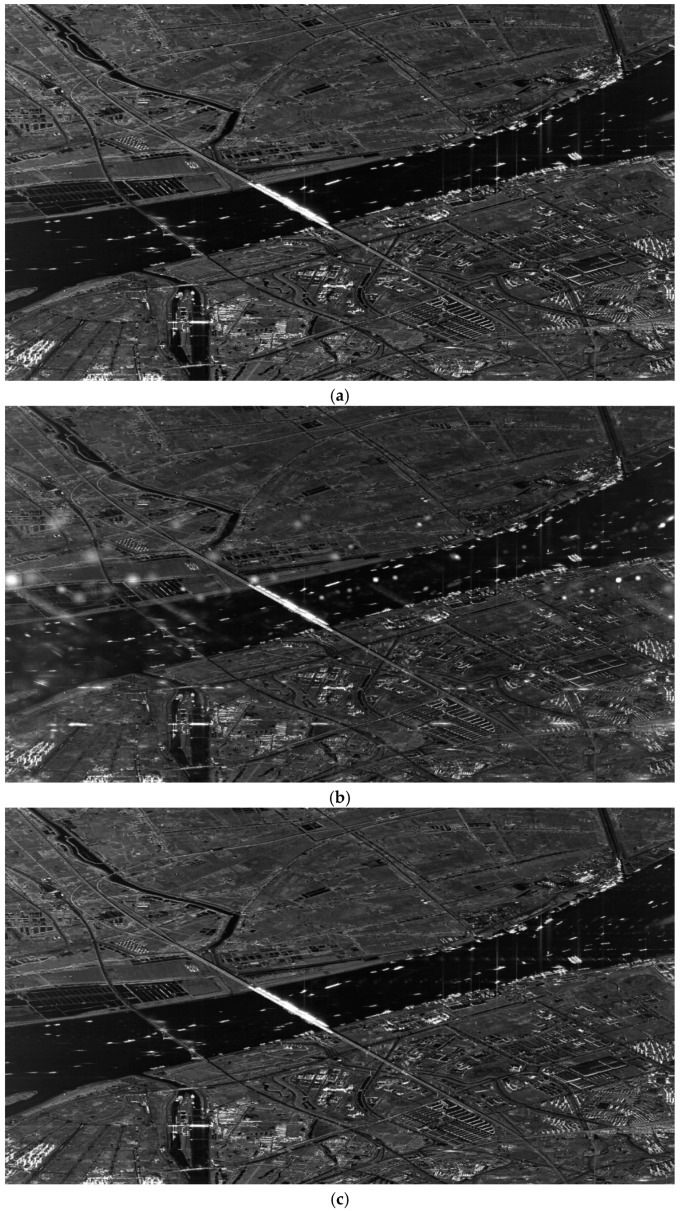
Experiments on GF-3 data. (**a**) Result of conventional two-step processing approach without data-preprocessing. (**b**) Result of conventional two-step processing approach with data-preprocessing. (**c**) Result of modified two-step processing approach (based on modified sinc interpolation) with data-preprocessing.

In Figure 6a, an ideal image whose resolution is about 0.5 m is shown without any alteration. After that, data-preprocessing is employed to form periodical nonuniformly sampled data, and the image focused by the conventional two-step processing approach is shown in Figure 6b. Figure 6c shows how the same image would be improved by the modified sinc interpolation.

As is apparent, after data-preprocessing, the targets are still present in image, but the imaging quality is significantly affected by azimuth ambiguities, which is illustrated in Figure 6b. After the application of modified sinc interpolation, as shown in Figure 6c, the azimuth ambiguities decrease significantly, which makes the image quality like that of Figure 6a.

**Figure 7:**
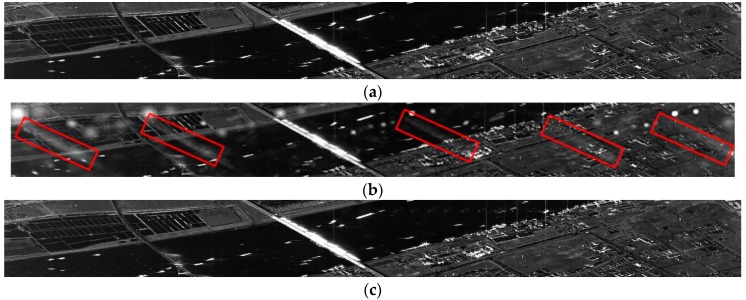
Sub-area of the train in Figure 6. (**a**) Result of conventional two-step processing approach without data-preprocessing. (**b**) Result of conventional two-step processing approach with data-preprocessing. (**c**) Result of modified two-step processing approach (based on modified sinc interpolation) with data-preprocessing.

To further figure out the problems of the false targets, which are caused by nonuniform sampling and spectrum folding effects, some sub-areas are extracted from Figure 6 and shown in Figure 7. In Figure 7a, there is no false targets of the train which is obviously visible on the river. In Figure 7b, there are several false targets of the train marked by red frames. It can be seen that, by using data-preprocessing described above, the conventional two-step processing approach induces the false targets arising Besides, the false targets are invisible in Figure 7c. This means that the azimuth ambiguities of strong targets can be degraded apparently by the modified sinc interpolation.

**Figure 8:**
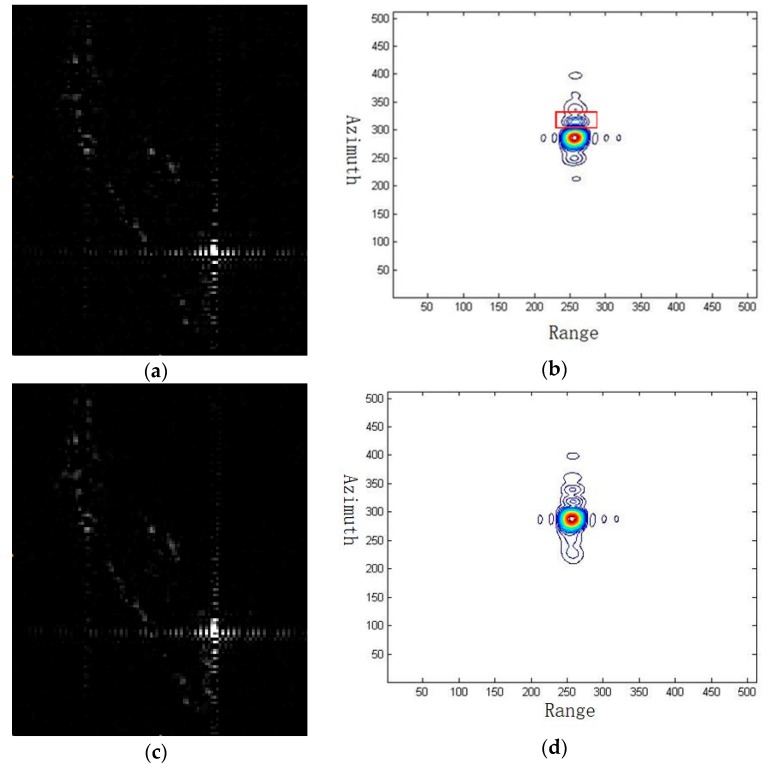
Nonstandard target result chosen from Figure 6. (**a**) Image by conventional two-step processing. (**b**) Contour by conventional two-step processing. (**c**) Image by modified two-step processing. (**d**) Contour by modified two-step processing.

To better clarify the effect of the modified sinc interpolation on point target, we choose a nonstandard point target on a ship, which is shown in Figure 8.

From Figure 8, it is clearly seen that the conventional two-step processing approach leads to sidelobe rising as marked by the red frame in Figure 8b. To further demonstrate the imaging qualities of this point target, Table 4 compares both the peak sidelobe ratio (PSLR) and the integrated sidelobe ratio (ISLR) of the conventional and the modified two-step processing approaches. It is clear that the performance of the proposed method is better than that of the traditional one as the PSLR and ISLR are enhanced a little bit.

## 5. Conclusions

This paper focuses on the signal processing for spotlight SAR with continuous PRI variation and very high azimuth resolution. A modified sinc interpolation is proposed in this paper to reconstruct a uniformly sampled signal in a time domain. As the kernel weight decreases with the distance away from the interpolation point, the computational cost of the modified sinc interpolation is far less than NUDFT by choosing a suitable kernel length. Combined with the traditional two-step processing approach, the modified sinc interpolation is proposed to reconstruct the nonuniformly sampled raw data after azimuth deramping, which further expands the applicability of the two-step processing approach. The simulation results verify the validity of the proposed method. The modified sinc interpolation has better performance in dealing with a nonuniformly sampled signal than the conventional sinc interpolation; besides, the modified sinc interpolation reduces less computational resources compared to NUDFT. In fact, the modified sinc interpolation can also be applied to other nonuniform sampling SAR systems, such as azimuth multi-channel SAR, to improve the focusing performance.

## Figures and Tables

**Table 1:** SAR parameters of two types of PRI variation.

Parameter	Value
Wavelength (m)	0.0312
Orbit height (km)	1100
Look angle (degree)	49
PRF span (Slow change) (Hz)	3243–3355
PRF span (Fast change) (Hz)	3243–5964
Pulse duration (µs)	30
Pulse number in a sequence period (Slow change)	110
Pulse number in a sequence period (Fast change)	64
Nominal azimuth Resolution	0.1
Illuminated area (km)	8

**Table 2:** Complexity of calculation.

Methods	Complex Multipication	Complex Additons	Total
NUDFT	*Na* ^2^	*Na* × (*Na* − 1)	*Na* × (2*Na* − 1)
Modified sinc interpolation	*Na* × *L*	*Na* × (*L* − 1)	*Na* × (2*L* − 1)

**Table 3:** False targets level of the point targets of Figure 4 and Figure 5.

Variation of PRI	Algorithm	Near-Range Target	Mid-Range Target	Far-Range Target
Slow linear variation of PRI	FFT	−20.56 dB	−48.44 dB	−22.11 dB
	NUDFT	−71.56 dB	−72.91 dB	−72.57 dB
	Traditional sinc	−49.38 dB	−50.17 dB	−49.87 dB
	Modified sinc	−67.22 dB	−66.89 dB	−71.61 dB
Fast linear variation of PRI	FFT	−12.33 dB	−31.98 dB	−12.04 dB
	NUDFT	−54.03 dB	−54.25 dB	−54.57 dB
	Traditional sinc	−26.05 dB	−26.11 dB	−26.02 dB
	Modified sinc	−56.48 dB	−53.36 dB	−54.95 dB

**Table 4:** Results of a nonstandard point target.

Method	PSLR	ISLR
Conventional two-step processing approach	−10.954 dB	−10.067 dB
Modified two-step processing approach	−13.844 dB	−12.130 dB
